# Radiologic Evaluation of Compressive Osseointegration for the Fixation of Reconstruction Prostheses after Tumor Resection

**DOI:** 10.1155/2015/513939

**Published:** 2015-07-22

**Authors:** Manol Lazarov, Thomas De Bo, Bart Poffyn, Gwen Sys

**Affiliations:** Department of Orthopaedics, University Hospital Ghent, De Pintelaan 185, 9000 Ghent, Belgium

## Abstract

*Objective*. In pursuance of thoroughly understanding and facilitating the evaluation of the radiological changes in the preloaded bone by Compliant Pre-Stress osseointegration (Compress Biomet, Warsaw, Indiana) a new staging method was created depicting four stages. *Methods*. Two cohorts (10 and 17 patients resp., not-receiving and receiving chemotherapy) were compared in terms of progression of osseointegration. Based on the changes at the bone-metal interface seen on röntgenorgrams four stages were defined: stage 0: immediate postoperative status, no ingrowth, or noncalcified callus; stage 1: early mineralization, calcified callus; stage 2: mature mineralization; and stage 3: hypertrophy at the level of the pins. *Results*. There were no significant differences between the two cohorts. Group 2, which was significantly younger than group 1 (*p* < 0.001), presented a delayed initial rate of bone formation and reached stage 1 at 6 months instead of 3 months like group 1. The children from the group 2 demonstrated a visible rebound ingrowth. *Conclusion*. Despite the fact that the staging fails to demonstrate a statistical difference, it is rather simple and can be used for future studies.

## 1. Introduction

Due to the improved imaging techniques and chemotherapy regimens nowadays, limb sparing procedures become the mainstay of treatment of primary bone tumors [1]. Unfortunately, the long term results of classic long-stem endoprosthetic reconstructions (cemented or uncemented) are poor due to loosening and/or stress shielding, displaying a survival rate of 83 percent at five years and only 67 percent at ten years [2]. As the patient population is generally young, major revisions are required to restore a stable fixation, associated with additional bone loss [3, 4]. Stems with a coated collar allowing active bone ingrowth proved to provide a better fixation on the long term [5–7]. Dynamic compression fixation (Compress) of tumor prostheses was proposed as an alternative to long stems, aiming to improve the long term survivorship of the implant through active osseointegration. By using a short traction bar the ongoing compressive force generated by Belleville washers stimulates osseointegration at the bone-prosthetic interface [8, 9] and promotes hypertrophy of the loaded bone. In literature currently no radiographic scoring system is available to evaluate the ingrowth and the bone hypertrophy in a straightforward way. For instance, the ISOLS radiographic scoring system is not applicable to this kind of fixation [10]. For the purpose of easier and proper radiologic evaluation and comparison, a new staging method was developed.

## 2. Study Design and Methods

Between 2006 and 2014 27 patients with distal third femoral replacements only were included in this study. All interventions were performed in a single university hospital by the same orthopedic surgeon (GS). A reconstruction prosthesis (OSS, Biomet, Warsaw, Indiana) was used in all patients, fixed to the bone by a Compress device. All patients had an initial resection of a bone tumor (19 bone sarcomas, 2 giant cell tumors of bone, and 2 soft tissue sarcomas affecting the bone). Ten patients (group 1) did not receive chemotherapy at least 1 year before the surgical intervention. Sixteen patients (group 2) received neoadjuvant and adjuvant chemotherapy according to the EURAMOS [11] and one Euro-Ewing protocol [12].

The treatment was coordinated by a multidisciplinary oncological team specialized in sarcoma treatment. Demographics and diagnoses are presented in Table 1.

The mean follow-up for patients enrolled in the study was 36 months (median 34 months, range 15–66 months). During the study one patient had an amputation because of full thickness flap failure at 2 months. Another one developed early postop infection treated with debridement and irrigation. The age distribution was 9–68 years mean 27; there were 15 females and 12 males. Data were collected up to 18 months postoperatively. The mean resection length was 15 cm (median 16 cm, range 8–26 cm). Only cases of femoral osseointegration were included, in Table 2.

Radiological assessment (standard AP and lateral view) took place immediately after the operation. Afterwards office visits were scheduled for clinical evaluation and radiographs at 6 weeks, 3, 6, 9, 12, 15, and 18 months after surgery. No patients were recalled specifically for the study. The radiographs were taken before the consultation and afterwards coded without names and dates by the study nurse. The radiographic evaluation was performed independently by two orthopedic surgeons according to the new staging system and was repeated three times.

**Figure 1:**
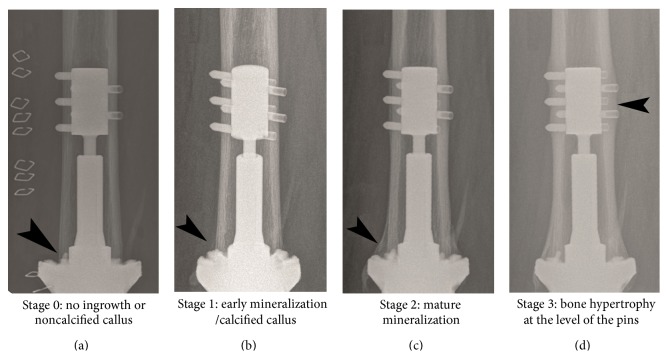
Radiographic stages of osseointegration according to the new assessment system.

For the staging system four radiological stages were defined: stage 0: immediate postoperative status, no ingrowth, or noncalcified callus; stage 1: early mineralization, calcified callus; stage 2: mature mineralization; and stage 3: hypertrophy at the level of the pins, as in Figure 1.

The Medical Ethics Committee granted approval for the study and informed consent was obtained from all the patients or their parents.

### 2.1. Surgical Technique

After en bloc removal of the tumor, a standard technique for prosthetic reconstruction was used as recommended by the manufacturer [8, 9]. Shortly, during the preparation of the diaphyseal bone special attention was taken to protect the blood supply in the bone-prosthesis interface region by minimal reaming and protecting the periosteal membrane. Subsequently the anchor plug and traction bar were secured to the cortical bone via transverse pins. The spindle containing the Belleville washers was attached to the traction bar by means of a nut, resulting in a compression of the spindle to the bone. The compression force used, depending on the cortical width, varied between 600 and 800 lb. (181–363 kg) and serves to promote bone ingrowth at the bone-prosthetic interface and to induce bone hypertrophy according to Wolff's Law. Two or three antirotational pins were used to avoid loosening of the device by rotational forces. These pins were available since the end of 2011, not before.

Rehabilitation encompassing active-assisted and gentle active range of motion among with muscle strengthening exercises was initiated immediately after surgery. A strict nonweight bearing protocol was recommended until the radiographs showed early callus mineralization (stage 1), after which partial weight bearing was allowed and gradually increased. Activities creating rotational torque such as kneeling, crossing the legs, and squatting were obviated but cycling and swimming were allowed. Repetitive or high impact activities were strictly prohibited.

### 2.2. Statistical Analysis

Student's *t*-test was performed using SPSS1 Version 22, 2013 (SPSS Inc., Chicago, IL, USA). Inter- and intraobserver variation was tested with Intraclass Correlation Coefficient (ICC 2.1) evaluating blinded radiographs.

The null hypothesis was that age, location, and chemotherapy did not affect ingrowth significantly. A *p* value less than 0.05 was considered to be statically significant.

## 3. Results

The study included a total of 27 patients who completed the study. Group 2 was statistically younger than group 1 (*p* = 0.0003811). As we mentioned above a radiological assessment took place at every visit at preset time intervals (0 and 6 weeks and 3, 6, 9, 12, 15, and 18 months postoperatively). When defining the ingrowth stages there was neither interobserver nor intraobserver variability, ICC 2.1 consistency of 1.

**Figure 2:**
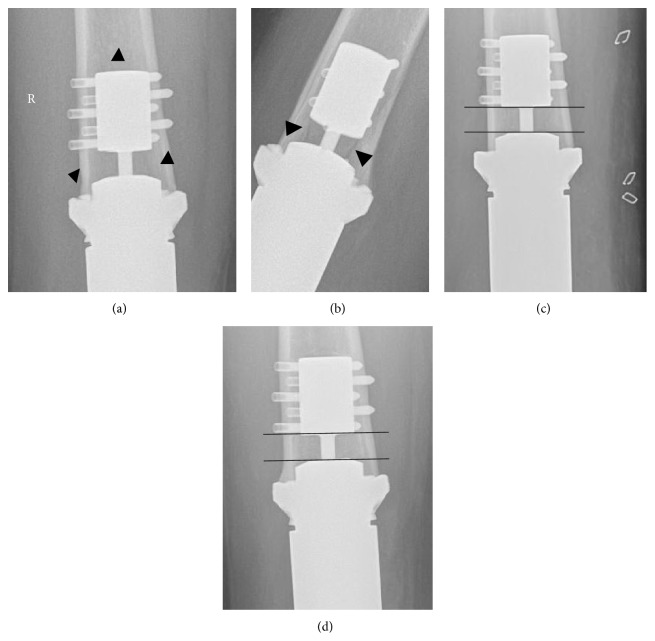
(a) and (b) Radiolucent line in the compress area after falling 1 y postop, (c) and (d) development of angulation and loss of distance between the anchor plug and the spindle can be observed after fracture (d) healing when compared to the prefracture X-ray (c). At 18 months stage 3 has not been reached (d).

Furthermore during the radiographic evaluation signs of loosening were sought as previously suggested in literature [6] fracture, transverse pin migration, and progressive radiolucency at the bone-prosthetic interface. The length of the visible part of the traction bar was measured and also the angle between the anchor and the spindle in order to exclude collapse, indicating loss of compression force. A fracture line was seen in this area in one patient after a fall at home one year after surgery. A diminished distance as well as an angulation between the spindle and the anchor plug was noted (Figure 2). The patient was closely observed: no fracture displacement was seen after one year, but the radiographic assessment showed no progression from stage 2 to stage 3.

For all patients stage 1 was generally reached at 6 months, stage 2 at 9 months, and stage 3 at 12 months. At 18 months all patients but three had reached stage 3. One of them has been just described above (Figure 2). Another one from group 2 showed progression and received longer chemotherapy and resection of pulmonary metastases. Interestingly, the third patient who was revised for an infected megaprosthesis using a Compress system reached stage 3 ingrowth by 18 months despite reinfection and two-time debridement. In one patient from group 1 palliative chemotherapy was started after 16 months after reaching stage 3. From the patients with remaining growing potential there was one child who had a revision of a long stem of the femur who did not receive chemotherapy.

There was neither stress shielding nor osteonecrosis nor transverse pin migration in this study. Other complications irrelevant to the study were also noted.

**Figure 3:**
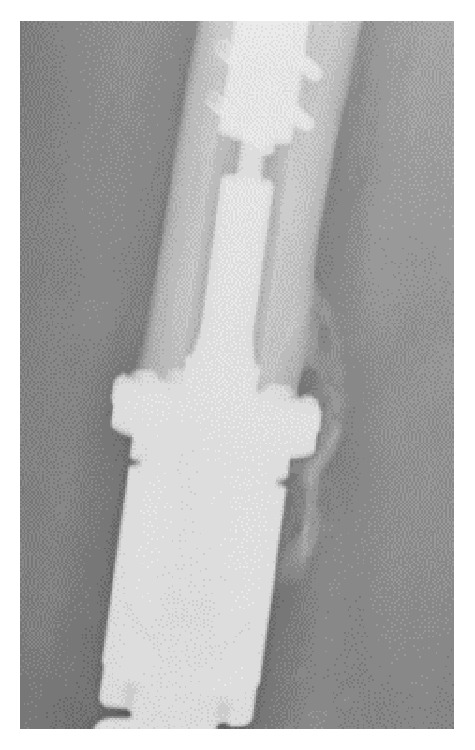
Heterotopic ossification seen at 6 months postop.

In our institution ultrasound is used for the local follow-up aiming at detection of local tumor recurrence in the proximity of large prosthetic implants. During the early stages an intense hypervascular aspect at the bone-prosthetic interface is observed. This phenomenon is also very distinct in a Technetium bone scan. Furthermore, callus formation should not be confused with heterotopic ossification, as seen in Figure 3.

The data were scrutinized in order to evaluate the effect of age and chemotherapy on the time interval on which patients reach the next stage of ingrowth. Statistically there was no significant difference between the two groups (*p* > 0.05). Eventually the patients with still growing potential from group 2 were compared with the rest of the group and also group 1. A cut-off of 15 years at the time of surgery was chosen. The *p* values were still above 0.05. The null hypothesis was not rejected.

**Figure 4:**
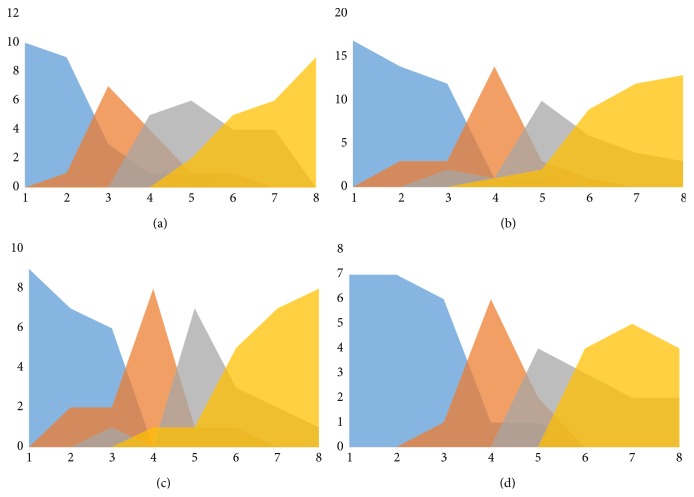
Four graphs representing the effect of chemotherapy and age on the stage. (a) Patients not receiving chemotherapy. (b) Patients receiving chemotherapy demonstrate a long plateau stage 0 with delayed onset of bone formation at 6 months. When comparing the children and adults from this group (charts (c) and (d)) a rebound ingrowth and shorter period between stages 2 and 3 and reaching stage 3 earlier are observed.

When the effect of chemotherapy and age were charted, it seemed that patients receiving chemotherapy generally reached stage 1 at a later time point (6 months instead of 3 months). When children were compared to young adults in the chemotherapy group, it seemed that children displayed a rebound ingrowth after cessation of chemotherapy resulting in a shorter period between stages 2 and 3 and reaching stage 3 at an earlier time point, as shown in Figure 4.

## 4. Discussion

Dynamic compressive fixation of tumor prosthesis is a method introduced to the orthopedic surgery aiming to achieve biomechanical stable and durable implants while stimulating osseointegration. In literature there is currently no universal method describing bone remodeling and ingrowth following Compress fixation. Fracture callus measurement is also not an easy task [13] and is therefore not routinely used in daily practice. Avedian et al. [14] used digital calipers on standardized radiographs to measure the bone width. In our opinion this method is cumbersome and hard to reproduce when it comes to a multicenter research trial.

For this reason a new algorithm was proposed by the senior author of the group, which seems to be quite easy and straightforward. However, correct assessment of osseointegration also has a learning curve. When the ultrasound specialist is not familiar with the device, the image obtained during the development of the uncalcified callus may be misdiagnosed as a local recurrence due to the hypervascular aspect at the bone-prosthetic interface. The same is true for a Tc bone scan. Furthermore, callus formation should not be confused with heterotopic ossification as seen.

The authors have not observed the development of bone hypertrophy before stages 1 (early mineralization) and 2 (mature mineralization) have been reached. Apparently bone hypertrophy, a phenomenon observed at the periosteal side as well as at the endosteal side, is a slow continuous process, possibly starting at the same moment as the osseointegration, but this is hard to discern on radiographs. Full development of stage 3 was seen only in the femur but not in the tibia or in the humerus, which is a phenomenon we cannot explain.

We have observed a cessation of bone hypertrophy towards stage 3 after loss of compression force due to a fracture. This indicates the usefulness of a staged radiographic assessment for studying the influence of certain factors such as chemotherapy on the bone formation at the prosthetic interface. Although it is a small study and far from representative, we were able to demonstrate that, after the cessation of chemotherapy, progressive bone ingrowth is initiated. The negative effect of chemotherapy on bone metabolism has been well demonstrated in the literature. The impact of chemotherapy on graft healing was described in animal [15–18] and human [19] studies showing an increase of delayed union.

When it comes to osseointegration Avedian et al. observed a decrease in the absolute amount and the rate of bone hypertrophy in the group receiving chemotherapy. In our study we could demonstrate a trend towards a slower onset of stage 1 in patients receiving chemotherapy, but eventually almost all patients reach stage 3. Lack of significance is our opinion due to limited number of patients and diversity of the groups. Different local factors such as blood supply, preserving the intraosseous and small periosteal vessels, irrigation avoiding thermal necrosis, and soft tissue coverage are crucial elements during surgery. Osteonecrosis of the bone at the bone-Compress interface can be an important complication as mentioned by Healey et al. [7], but this phenomenon was not observed in our patient group.

## 5. Conclusion

This paper describes a new methodology for the radiographic assessment of compressive osseointegration for the fixation of reconstruction prostheses after tumor resection. The application of the method is easy, allowing international comparison of prosthetic survival after Compress implantation. The authors propose to use this evaluation method also for studying factors delaying or preventing bone hypertrophy at the prosthetic interface such as age and chemotherapy regimen, causing prosthetic failure.

## Figures and Tables

**Table 1:** Demographic information.

	Age at	Gender	Diagnosis	Bone	Location	Type
	surgery			soft tissue	bone resection	
1	10	Female	3,5 years tumor-free, leg length discrepancy	Bone	Femur	Total knee
2	31	Female	14 year tumor-free, femoral loosening	Bone	Femur	Total knee
3	33	Male	9 year tumor-free, infection	Bone	Femur	Total knee
4	41	Male	22 y tumor-free, loosening tumorprothese knee replacement		Femur	Total knee
5	40	Female	Leiomyosarcoma	Bone	Femur	Total knee
6	65	Female	Leiomyosarcoma	Soft tissue	Femur	Total knee
7	38	Male	Giant cell	Bone	Femur	Total knee
8	63	Female	Giant cell	Bone	Femur	Total knee
9	57	Female	Chondrosarcoma	Bone	Femur	Total knee
10	68	Male	Chondrosarcoma	Bone	Femur	Total knee

1	7	Male	Osteosarcoma	Bone	Femur	Total knee
2	9	Female	Osteosarcoma	Bone	Femur	Total knee
3	11	Male	Osteosarcoma	Bone	Femur	Total knee
4	11	Female	Osteosarcoma	Bone	Femur	Total knee
5	12	Male	Osteosarcoma	Bone	Femur	Total knee
6	14	Female	Osteosarcoma	Bone	Femur	Total knee
7	14	Female	Osteosarcoma	Bone	Femur	Total knee
8	14	Male	Osteosarcoma	Bone	Femur	Total knee
9	15	Female	Osteosarcoma	Bone	Femur	Total knee
10	16	Male	Osteosarcoma	Bone	Femur	Total knee
11	16	Female	Osteosarcoma	Bone	Femur	Total knee
12	17	Female	Osteosarcoma	Bone	Femur	Total knee
13	19	Male	Osteosarcoma	Bone	Femur	Total knee
14	19	Female	Osteosarcoma	Bone	Femur	Total knee
15	22	Male	Ewing sarcoma	Bone	Tibia	Total knee
16	26	Male	Osteosarcoma	Bone	Femur	Total knee
17	30	Female	Osteosarcoma	Bone	Femur	Total knee

**Table 2:** The summary of the demographic characteristics of the two groups.

Characteristic	Value
Number of patients	27
Compress fixed	27
Follow-up	Mean 36 months (range 15–66)
Male	12 (45.5%)
Female	15 (55.5%)
Age at surgery	
Nonchemo	Mean 45, median 41 (range 10–65)
Chemo	Mean 16, median 15 (range 9–30)
*p* value	0.000377663
Length of resection	
Nonchemo	Median 13.8 (range 8–23.5)
Chemo	Median 15.3 (range 8–23.5)
*p* value	0.303972
Children (growing potential at surgery)	9 (33.3%)
Adults treated with chemotherapy	8 (29.6%)
Adults not treated with chemotherapy	10 (37%)
Diagnosis	
Osteosarcoma	16 (59.2%)
Ewing sarcoma	1 (3.7%)
Chondrosarcoma, giant cell, and leiomyosarcoma	2 (7.4%)
Revision	
Loosening	2 (7.4%)
3,5 years tumor-free, leg length discrepancy	1 (3.7%)
9 year tumor-free, infection	1 (3.7%)
